# Modeling the therapeutic efficacy of *NFκB* synthetic decoy oligodeoxynucleotides (ODNs)

**DOI:** 10.1186/s12918-018-0525-6

**Published:** 2018-01-30

**Authors:** Zhipeng Wang, Davit A. Potoyan, Peter G. Wolynes

**Affiliations:** 1 Center for Theoretical Biological Physics, Rice University, Houston, 77005 TX USA; 2 Department of Chemistry, Rice University, Houston, 77005 TX USA; 3 Department of Physics and Astronomy, Rice University, Houston, 77005 TX USA; 4 Present Address: Department of Chemistry, Iowa State University, Ames, 50011 IA USA; 5 Present Address: Genentech Inc. 350 DNA Way, South San Francisco, 94080 CA USA

**Keywords:** NF *κ*B synthetic decoy ODNs, Systems pharmacology, Systems biology, Therapeutic efficacy

## Abstract

**Background:**

Transfection of NF *κ*B synthetic decoy Oligodeoxynucleotides (ODNs) has been proposed as a promising therapeutic strategy for a variety of diseases arising from constitutive activation of the eukaryotic transcription factor NF *κ*B. The decoy approach faces some limitations under physiological conditions notably nuclease-induced degradation.

**Results:**

In this work, we show how a systems pharmacology model of NF *κ*B regulatory networks displaying oscillatory temporal dynamics, can be used to predict quantitatively the dependence of therapeutic efficacy of NF *κ*B synthetic decoy ODNs on dose, unbinding kinetic rates and nuclease-induced degradation rates. Both deterministic mass action simulations and stochastic simulations of the systems biology model show that the therapeutic efficacy of synthetic decoy ODNs is inversely correlated with unbinding kinetic rates, nuclease-induced degradation rates and molecular stripping rates, but is positively correlated with dose. We show that the temporal coherence of the stochastic dynamics of NF *κ*B regulatory networks is most sensitive to adding NF *κ*B synthetic decoy ODNs having unbinding time-scales that are in-resonance with the time-scale of the limit cycle of the network.

**Conclusions:**

The pharmacokinetics/pharmacodynamics (PK/PD) predicted by the systems-level model should provide quantitative guidance for in-depth translational research of optimizing the thermodynamics/kinetic properties of synthetic decoy ODNs.

**Electronic supplementary material:**

The online version of this article (doi:10.1186/s12918-018-0525-6) contains supplementary material, which is available to authorized users.

## Background

The transcription factor *NFκB* is a central regulator for many genes in eukaryotic cells, orchestrating the immune response to inflammation, apoptosis, proliferation, differentiation and many more activities [1–4]. *NFκB* represents a family of dimeric proteins. In our study the term *NFκB* refers specifically to p65-p50 heterodimers, which are found widely in most cell types. While induced activation of *NFκB* plays a pivotal role in regulating immune and inflammatory responses, constitutive *NFκB* activation is observed in many pathologies [2, 4]. Such constitutive activity is widely considered as a major causal event for many human diseases, including chronic inflammation, auto-immune diseases and cancer etc [5–8]. In the clinic, inhibition of *NFκB* activation has shown to be a promising treatment strategy for *NFκB*-related diseases [9–11].

*NFκB* Synthetic decoy oligodeoxynucleotides (ODN), which are consensus double-stranded DNA segments mimicking the *NFκB* DNA binding sites, have already shown promising efficacy in inhibiting activation of *NFκB* [12–15], by simply binding to free *NFκB* to block interactions with its binding sites on the genome. Clinical studies showed that synthetic *NFκB* decoy ODNs lead to minimal side effects and display less toxicity than other treatment methods [13–15]. Although there has been much progress using innovative and bio-compatible methods to deliver *NFκB* decoy ODNs into the cell nucleus [16–18], there is still a limited quantitative understanding of the pharmacology of *NFκB* decoy ODNs. In this work, we aim to understand how *NFκB* synthetic decoy ODNs affect the systems biology of the entire biological network of *NFκB* signaling, and if network models can provide quantitative information about the therapeutic benefits of *NFκB* synthetic decoy ODNs.

**Fig. 1:**
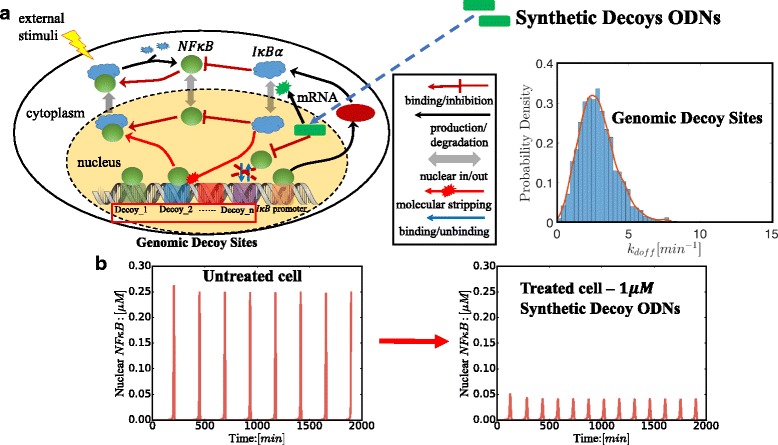
**a** Minimal model of *NFκB* signaling networks and the therapeutic strategy of *NFκB* synthetic decoy ODNs. Protein-binding Microarrays (PBMs) experiments indicate that the unbinding rates of genomic decoy sites (indicated in Fig. 1. as Decoy _1, 2…) follow a distribution plotted in Fig. 1a. **b** Oscillatory temporal dynamics of nuclear free *NFκB* and inhibitory effect of synthetic decoy ODNs

Systems pharmacology is the application of systems biology principles to the field of pharmacology, and has emerged lately as a quantitative approach to study the effect of a drug [19]. Instead of investigating the interactions between the drug and its target molecules, systems pharmacology considers the effect of a drug as the result of the network of interactions the drug may have with other components in the complex biological system. In this paper, we set up a systems pharmacology model for the influence of *NFκB* decoy ODNs, based on recent advances in modeling the *NFκB* signaling networks [20–22]. To this end, It is essential to map out the network of interactions/chemical reactions of the relevant biomolecules. As illustrated in Fig. 1a, the minimal model of the *NFκB* regulatory network includes continuous extracellular stimulation which leads to constitutive activation of *NFκB*, binding/unbinding of *NFκB* to the *IκB* promoter, transcription of the *IκBα*-encoding gene to mRNA, and translation of mRNA to *IκBα* proteins. It also includes binding/unbinding of *NFκB* to both genomic decoy sites and synthetic decoy ODNs. Here we define genomic decoy sites as all of the *NFκB* binding sites on the genome except for the *IκB* promoter, while synthetic decoy ODNs are artificially-synthesized DNA sequences mimicking the *NFκB* binding sites.

A full description of the model setup is presented in the Methods section. The network contains a time-delayed negative feedback loop in which translated *IκBα* proteins translocate from the cytosol to the nucleus where they remove nuclear *NFκB*. The resulting time delay leads to oscillatory temporal dynamics of free nuclear *NFκB* (As shown in Fig. 1b) [20, 21, 23]. The model also contains the recently-discovered molecular stripping process [24, 25], in which *IκBα* induces the active dissociation of *NFκB* from binding sites. The binding affinities of genomic decoy sites are distributed according to values inferred from Protein Binding Microarrays(PBMs) experiments [26, 27], which leads to a range of unbinding kinetic rates. The associated kinetic rates for the *IκB* promoter and related chemical reactions are based on experimental results [20, 21, 23].

The therapeutic role of synthetic decoy ODNs is to bind to nuclear *NFκB* molecules to attenuate their interactions with functional genomic binding sites. In the field of medicine, “therapeutic efficacy” is usually defined as the actual beneficial change demonstrated by a drug under a certain dose, and it is usually measured in a well-designed clinical trial [28]. In this paper we follow a pharmacology-based interpretation and define “therapeutic efficacy” of synthetic *NFκB* decoy ODNs as the inhibitory capacity of nuclear *NFκB* activity [29]. The present model allows us to quantify the dependence of the therapeutic efficacy of *NFκB* synthetic decoy ODNs on their dose level, their binding/unbinding kinetic rates and the rate of molecular stripping. The model also predicts the pharmacokinetics and pharmacodynamics (PK/PD) of synthetic decoy ODNs under different degradation rates arising from nuclease activity [30]. These results should provide some quantitative guidance for translational researchers and drug developers for the design of therapies based on *NFκB* decoy ODNs.

## Methods

In this paper, we set up both deterministic and stochastic kinetic models to study the systems pharmacology of *NFκB* synthetic decoy ODNs. The aim of our models is to quantify how the synthetic decoy ODNs change the dynamics of nuclear *NFκB* and other relevant species in the regulatory network. The deterministic kinetic model is based on a set of differential equations capturing the collective behavior of the network, which can be used to predict population-level dynamics of the species in the network when they have large copy numbers; while the stochastic model is based on a master equation which captures changes of the probability of any particular micro-state of the *NFκB* regulatory network. It can be used to predict single-cell level stochastic dynamics of the species in the network when they have small copy numbers. Both classes of models for the regulatory network of *NFκB* have been extensively studied [3, 20, 23, 31, 32]. Here we want to highlight the novel elements in our model. A high-level description of our minimal model is illustrated in Fig. 1a. The present models contain both *NFκB* genomic decoy sites and synthetic decoy ODNs, the degradation of synthetic decoy ODNs, as well as the binding/unbinding reactions of *NFκB* to the synthetic decoy ODNs. They also incorporates *IκB*-induced bimolecular molecular stripping of *NFκB* from bound sites on the genome and synthetic decoy ODNs in addition to the spontaneous unimolecular dissociation that was used alone in earlier models [3, 31]. The names of the molecular species involved in the network, their associated reactions and kinetic coefficients are listed in Tables 1 and 2. We will elaborate the deterministic model and the stochastic model respectively in the following sections.

**Table 1:** Chemical Reactions for *IκBα*/*NFκB* regulatory circuit with *NFκB* synthetic decoy ODNs. The parameters of the feedback cycle originate from the work of Hoffmann et al. [20] while the ranges of values for specific binding/unbinding rates come from binding microarray data [26] and in vitro kinetic measurements [24, 51]

Reactions	Rate coeff	Values
*D*_*U*_+*N*_*n*_→*D*_*B*_	*k*_*don*_	10 *μM*^−1^*min*^−1^
*D*_*B*_→*D*_*U*_+*N*_*n*_	*k*_*doff*_	$\sim LogNormal (\Delta \hat {G}, \sigma ^{2})$
*AD*_*U*_+*N*_*n*_→*AD*_*B*_	*k*_*son*_	10 *μM*^−1^*min*^−1^
*AD*_*B*_→*AD*_*U*_+*N*_*n*_	*k*_*soff*_	[0.02−1] *min*^−1^
*AD*_*U*_⇒*∅*	*λ*_*s*_	[0−0.02] *min*^−1^
*AD*_*B*_⇒*∅*	*λ*_*s*_	[0−0.02] *min*^−1^
*OFF*+*N*_*n*_→*ON*	*k*_*on*_	10 *μM*^−1^*min*^−1^
*ON*→*OFF*+*N*_*n*_	*k*_*off*_	1 *min*^−1^
*D*_*B*_+*I*_*n*_⇒*D*_*U*_+*NI*_*n*_	*k*_*s*_	[0−10] *μM*^−1^*min*^−1^
*ON*+*I*_*n*_⇒*OFF*+*NI*_*n*_	*k*_*s*_	[0−10] *μM*^−1^*min*^−1^
*AD*_*B*_+*I*_*n*_⇒*AD*_*U*_+*NI*_*n*_	*k*_*s*_	[0−10] *μM*^−1^*min*^−1^
*ON*⇒*ON*+*mRNA*	*k*_*tr*_	1.03 *μMmin*^−1^
*mRNA*⇒*mRNA*+*I*_*c*_	*k*_*tl*_	0.2448 *min*^−1^
*mRNA*⇒*∅*	*k*_*d*_	0.017 *min*^−1^
*I*_*c*_→*I*_*n*_	*k*_*in*_	0.018 *min*^−1^
*I*_*n*_→*I*_*c*_	*k*_*out*_	0.012 *min*^−1^
*N*_*c*_→*N*_*n*_	*k*_*Nin*_	5.4 *min*^−1^
*N*_*c*_+*I*_*c*_→*NI*_*c*_	*k*_*f*_	30 *μM*^−1^*min*^−1^
*NI*_*c*_→*N*_*c*_+*I*_*c*_	*k*_*b*_	0.03 *min*^−1^
*N*_*n*_+*I*_*n*_→*NI*_*n*_	*k*_*fn*_	30 *μM*^−1^*min*^−1^
*NI*_*n*_→*N*_*n*_+*I*_*n*_	*k*_*bn*_	0.03 *min*^−1^
*NI*_*c*_⇒*N*_*c*_	*α*	[0.10−0.55] *min*^−1^
*NI*_*n*_⇒*NI*_*c*_	*k*_*NIout*_	0.83 *min*^−1^

**Table 2:** Names of species and their numbers

Abbreviation	Full name
*D*_*B*_	Bound decoy site
*D*_*U*_	Unbound decoy site
*A* *D*_*B*_	Bound artificial decoy site
*A* *D*_*U*_	Unbound decoy site
*ON*	Active gene state
*OFF*	Inactive gene state
*I*_*n*_	Nuclear *IκBα*
*I*_*c*_	Cytoplasmic *IκBα*
*N*_*n*_	Nuclear *NFκB*
*N*_*c*_	Cytoplasmic *NFκB*
*N* *I*_*n*_	Nuclear *NFκB*−*IκBα* complex
*N* *I*_*c*_	Cytoplasmic *NFκB*−*IκBα* complex
*N*	Total number of *NFκB*: 10^5^
*Gene*≡*ON*+*OFF*	Total number of *Genes*: 1
*AD*≡*AD*_*B*_+*AD*_*U*_	Total number of artificial *Decoys*: [0−2×10^5^]
*D*≡*D*_*B*_+*D*_*U*_	Total number of natural *Decoys*: 2×10^4^

### Deterministic kinetic model for the systems pharmacology of synthetic decoy ODNs

The corresponding set of differential equations that constitutes our model is presented below.

1 $$ \begin{aligned} \frac{d [N_{n}]}{dt} = & k_{on}[N_{n}] [OFF] + k_{off} [ON] + k_{Nin} [N_{c}]\\ & - k_{fn} [N_{n}] [I_{n}] + k_{bn} [{NI}_{n}] \\ &-k_{don}[D_{U}]*[N_{n}] + k_{doff}[D_{B}] + k_{soff}[{AD}_{B}] \\ &- k_{son}[{AD}_{U}][N_{n}] \end{aligned}  $$

2 $$ \begin{aligned} \frac{d [I_{n}]}{dt} =& k_{Iin} [I_{c}] - k_{Iout}[I_{n}] - k_{fn}[N_{n}] [I_{n}] + k_{bn}[{NI}_{n}] \\ &-k_{s}[D_{B}] [I_{n}] - k_{s}[ON] [I_{n}] - k_{s}[{AD}_{B}][I_{n}] \end{aligned}  $$

3 $$ \frac{d [N_{c}]}{dt} = -k_{Nin} [N_{c}] - k_{f} [N_{c}] [I_{c}] + k_{b} [{NI}_{c}] + \alpha [{NI}_{c}]  $$

4 $$ \begin{aligned} \frac{d [I_{c}]}{dt} &= k_{tl} [mRNA] - k_{Iin} [I_{c}] + k_{Iout} [I_{n}]\\ & \quad - k_{f} [N_{c}] [I_{c}] + k_{b} [{NI}_{c}] \end{aligned}  $$

5 $$ \begin{aligned} \frac{d [{NI}_{n}]}{dt} =& k_{fn} [N_{n}] [I_{n}] - k_{bn} [{NI}_{n}] - k_{NIout} [{NI}_{n}] +\\ &+k_{s} [D_{B}] [I_{n}] + k_{s} [ON] [I_{n}] + k_{s}[{AD}_{B}][I_{n}] \end{aligned}  $$

6 $$ \frac{d [{NI}_{c}]}{dt}\! =\! k_{f} [N_{c}] [I_{c}] - k_{b} [{NI}_{c}] - \alpha [{NI}_{c}] + k_{NIout} [{NI}_{n}]  $$

7 $$ \frac{d[mRNA]}{dt} = k_{tr} [ON] - k_{d} [mRNA]  $$

8 $$ \frac{d [D_{B}]}{dt} = k_{don}[D_{U}] [N_{n}] - k_{doff}[D_{B}] - k_{s}[D_{B}] [I_{n}]  $$

9 $$ \begin{aligned} \frac{d [ON]}{dt} = k_{on}[N_{n}][OFF] - k_{off}[ON] - k_{s}[ON][I_{n}] \end{aligned}  $$

10 $$ \begin{aligned} \frac{d [{AD}_{B}]}{dt}& = k_{son}[{AD}_{U}] [N_{n}] - k_{soff}[{AD}_{B}]\\ & \quad - k_{s}[{AD}_{B}] [I_{n}] - \lambda_{s} [{AD}_{B}] \end{aligned}  $$

11 $$ \begin{aligned} \frac{d [{AD}_{U}]}{dt} & =- k_{son}[{AD}_{U}] [N_{n}] + k_{soff}[{AD}_{B}]\\ & \quad + k_{s}[{AD}_{B}] [I_{n}] - \lambda_{s} [{AD}_{U}] \end{aligned}  $$

In our model we assume there is a single *IκBα* promoter and 2×10^4^ genomic decoy sites for *NFκB* [33]. All of the *NFκB* genomic binding sites except for the *IκB* promoter are considered genomic decoy sites. The basis for this assumption are twofold: 1. Out of the at least 2×10^4^*NFκB* genomic binding sites discovered through Chip-seq experiments [33], only several hundred are promoter sites of genes whose expressions are regulated by *NFκB* [33, 34]. Therefore, most of the *NFκB* binding sites are non-specific with either unknown function or no functional role. 2. This paper focuses on a single module and the model can be simplified as the only activity of decoy ODNs is the sequestration of free *NFκB*. Thus, stoichiometry requires that [*ON*]+[*OFF*]=1 and [*D*_*B*_]+[*D*_*U*_]=2×10^4^. Dose is parameterized by the number of copies of synthetic decoy ODNs (AD). Finally, the total number of *NFκB* remains constant in the model and is set to a typical value for eukaryotes, which is approximately 10^5^. We set the cell volume so as to have a concentration of 1 *μM* which corresponds to 10^5^ copies of the *NFκB*, which is consistent to the range of eukaryotic cell volumes.

The set of ordinary differential equation (ODE) was solved using the integrator of real-valued variable-coefficient ODE solver, with fixed-leading-coefficient as implemented in Scipy library of python 2.7. The parameters were scanned on a fine grid within the ranges specified in each figure. Oscillatory dynamics was propagated for 3000 *min* discarding the first 500 *min* to eliminate any possible biases owing to initial conditions. We set the initial condition for our model to be: [*OFF*]=1,[*ON*]=0,[*D*_*B*_]=0,[*D*_*U*_]=2×10^4^,[*NI*_*c*_]=10^5^,[*AD*_*U*_]=*AD*,[*AD*_*B*_]=0 and the numbers of all the other chemical species are 0. However, the initial condition has no influence on the steady-state result because of the principle of limit cycle dynamics. The user can set up any initial condition according to the stoichiometry.

Transcription factor binding to DNA is commonly considered as a diffusion-limited step,hence we assume fast and uniform binding rates of *NFκB* to all of its binding sites including the *IκBα* promoter, genomic decoy sites and synthetic decoy ODNs (*k*_*on*_=*k*_*don*_=*k*_*son*_=10 *μM*^−1^*min*^−1^). For the *IκBα* promoter, the unbinding OFF rate *k*_*off*_ is set to be 1*min*^−1^ which generates an oscillation period consistent with experiments in Hela Cells [32]. We assume a normal distribution of binding free energies:$\Delta G_{b} \sim \mathcal {N}(\Delta \bar {G}, \bar {\sigma }^{2})$. The unbinding rates of natural decoys take a log-normal distribution:$\ln k_{doff} \sim \mathcal {N}\left (\Delta \hat {G},\sigma ^{2}\right)$, where $\Delta \hat {G} = \frac {\Delta \bar {G}}{k_{B}T} + \ln k_{don}$, and $\sigma ^{2} = (1/k_{B}T)^{2} \bar {\sigma }^{2}$. In order to perform computer simulation of the systems biology model, we approximate the log-normal distribution of *k*_*doff*_ by a histogram probability density estimator (See Additional files 1 and 2). In this work, we set $\Delta \hat {G} = 0$ and *σ*^2^=1 to mimic the results from protein binding microarrays (PBMs) experiments that reveal affinities of *NFκB* genomic binding sites.

### Stochastic kinetic model for the systems pharmacology of synthetic decoy ODNs

The stochastic dynamics of the *NFκB* regulatory network in the well stirred limit is governed by a master equation which relates the change of probability for a particular micro-state of the network to changes in the numbers of molecules, *z* as well as the occupancy state of the genomic binding sites, *σ*, where *z*={*z*_1_,*z*_2_,…,*z*_*N*_} is the vector containing numbers of molecules of each of the *N* chemical species in the network, and *σ*∈{0,1} is the binary variable representing the occupancy state of the genomic binding sites and synthetic decoy ODNs, with 0 indicating unoccupied state and 1 indicating occupied state.

12 $$ \begin{aligned} \dot{P}(z,\sigma) &= Q_{birth/death}(z \pm 1 \rightarrow z,\sigma)\\ & \quad - Q_{birth/death}(z \rightarrow z \pm 1,\sigma) \\ & \quad + Q_{bind/unbind}(z \rightarrow z', \sigma \rightarrow \sigma')\\ & \quad- Q_{bind/unbind}(z'\rightarrow z, \sigma' \rightarrow \sigma) \end{aligned}  $$

In this equation the first two terms (*Q*_*birth*/*death*_) denote the ingoing and outgoing probability fluxes via birth/death processes that change the total number of molecules (z) while the last two terms (*Q*_*bind*/*unbind*_) stand for probability fluxes caused by changes in the binary state (*σ*) of the binding sites (ON/OFF or bound/unbound). The initial condition and all the kinetic coefficients are the same as used in the deterministic model.

We employ a kinetic Monte Carlo scheme for solving the master equation of the minimal *NFκB* regulatory network [35] accounting for all of the discrete changes in the numbers of states of genomic binding sites (Tables 1 and 2). To quantify the temporal oscillatory dynamics, we calculate the normalized autocorrelation function of the free nuclear *NFκB*. We quantify the loss of coherence by calculating the dephasing time (*τ*_*ϕ*_) for the exponential decay ($\phantom {\dot {i}\!}e^{-t/\tau _{\phi }}$) fitted to the envelope of a periodic [*cos*(2*πt*/*T*)] normalized autocorrelation function (See Additional files 1 and 2). Here the dephasing time (*τ*_*ϕ*_) is defined as a quantity to represent the noise level in the stochastic network. Large dephasing time results in the slow decay rate of normalized autocorrelation function, indicating the small noise intensity and vice versa. Coherence is defined as the state where oscillation is sustained and is periodically consistent. Loss of coherence happens if noise is introduced into the oscillatory system. Detailed mathematical definition and derivations can be obtained from previous works [36, 37].

## Results

### *NFκB* synthetic decoy ODNs change both the steady-state nuclear *NFκB* concentration and the stochastic dynamics of the *NFκB* regulatory network

**Fig. 2:**
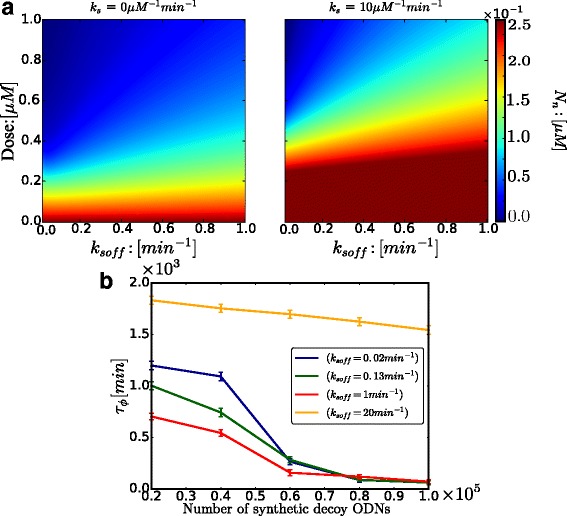
**a** Free nuclear *NFκB* peak concentrations as a function of Dose and unbinding rate (*k*_*soff*_) of synthetic *NFκB* decoy ODNs, under no molecular stripping (*k*_*s*_=0 *μM*^−1^*min*^−1^) and with molecular stripping (*k*_*s*_=10 *μM*^−1^*min*^−1^). **b** Dephasing Time (*τ*_*ϕ*_) of nuclear free *NFκB* with addition of synthetic decoy ODNs with different unbinding rates (*k*_*soff*_)

The therapeutic efficacy of *NFκB* synthetic decoy ODNs as a function of dose level and the unbinding kinetic rates *k*_*soff*_ is shown in Fig. 2a. Here the model does not account for the effect of the degradation of *NFκB* synthetic decoy ODNs, which is reported later. Figure 2a clearly illustrates that the efficacy is heavily dictated by both dose level and unbinding kinetic rates of *NFκB* synthetic decoy ODNs. Increasing the dose level monotonically decreases the steady-state amplitude of the nuclear *NFκB* oscillations, while increasing the unbinding kinetic rate *k*_*soff*_ attenuates the inhibitory efficacy. It is also clearly shown that molecular stripping increases the oscillatory amplitude of nuclear *NFκB* and decreases the therapeutic efficacy of decoy ODNs. With molecular stripping present, a higher dose is required to achieve the same level of therapeutic efficacy. Drug development therefore needs to take into account the negative influence on therapeutic efficacy of active-dissociation processes such as molecular stripping when tailoring dose and unbinding kinetic rates of drugs to achieve optimal efficacy.

Figure 2b illustrates the dephasing time of free nuclear *NFκB* (*τ*_*ϕ*_) as a function of the dose of *NFκB* synthetic decoy ODNs and their unbinding kinetic rates (*k*_*soff*_). We analyzed four different values for *k*_*soff*_, covering the range from slow unbinding rate(*k*_*soff*_=0.02 *min*^−1^) to fast unbinding rate(*k*_*soff*_=20 *min*^−1^). The temporal coherence of the network is relatively sensitive to the addition of *NFκB* synthetic decoy ODNs with unbinding rate *k*_*soff*_ falling in the resonant regime(*k*_*soff*_∼*k*_*off*_=1 *min*^−1^). The effect of adding slow *NFκB* synthetic decoy ODNs on the temporal coherence becomes weaker. Notably the temporal coherence is relatively insensitive to the addition of fast synthetic decoy ODNs. Understanding the role of noise in gene network dynamics is becoming increasingly important in disease therapeutics. These simulation results provide quantitative guidance for how decoys regulate the noise level of gene networks.

### Nuclease-induced degradation of synthetic decoy ODNs and PK/PD studies

One of the major limitations for the *NFκB* synthetic decoy ODNs is the degradation induced by intracellular nucleases [30]. In this study, only nuclease-induced degradation is considered. Owing to degradation, *NFκB* synthetic decoy ODNs can only be effective for a short period of time, which is defined as the duration of action in pharmacokinetics. In this study, we specifically define the duration of action of *NFκB* synthetic decoy ODNs to be the timespan during which they can inhibit the nuclear free *NFκB* activity to remain below 0.1 *μM*. This definition provides a consistent benchmark to describe the therapeutic effectiveness of *NFκB* synthetic decoy ODNs in a quantitative model.

**Fig. 3:**
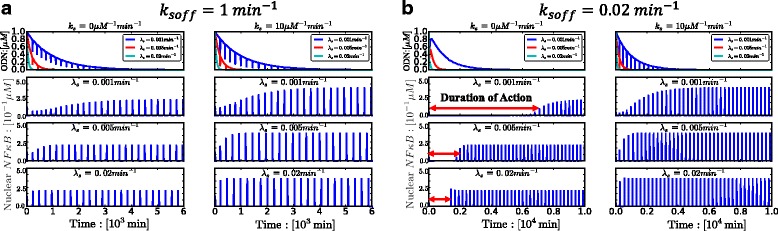
**a** Time courses of *NFκB* synthetic decoy ODNs and nuclear free *NFκB* concentrations under different degradation rates (*λ*_*s*_=0.001,0.005,0.02*min*^−1^) of synthetic decoy ODNs, under conditions of both no molecular stripping (*k*_*s*_=0 *μM*^−1^*min*^−1^) and with molecular stripping (*k*_*s*_=10 *μM*^−1^*min*^−1^). Unbinding rate of synthetic decoy ODNs is set to *k*_*soff*_=1 *min*^−1^. **b** Time courses of *NFκB* synthetic decoy ODNs and nuclear free *NFκB* concentrations under different degradation rates (*λ*_*s*_=0.001,0.005,0.02 *min*^−1^) of synthetic decoy ODNs, under conditions of both no molecular stripping (*k*_*s*_=0 *μM*^−1^*min*^−1^) and with molecular stripping (*k*_*s*_=10 *μM*^−1^*min*^−1^). Unbinding rate of synthetic decoy ODNs is set to *k*_*soff*_=0.02 *min*^−1^. The red arrows indicate the duration of action, during which the concentration of nuclear free *NFκB* does not exceed 0.1 *μM*

Figure 3a illustrates the time course of *NFκB* synthetic decoy ODNs concentrations and the time course of nuclear free *NFκB* concentrations with and without molecular stripping (*k*_*s*_=0 *μM*^−1^*min*^−1^ or *k*_*s*_=10 *μM*^−1^*min*^−1^) at different degradation rates (*λ*_*s*_=0.001,0.005,0.02 *min*^−1^). The dose is set to be 1 *μM* and the unbinding rate *k*_*soff*_ is set to be 1 *min*^−1^. The results clearly show that faster degradation rates lead to shorter duration of action while slower degradation rates, as one might expect, lead to longer duration of action. Figure 3a also highlights the effect of molecular stripping (*k*_*s*_=10 *μM*^−1^*min*^−1^) : nuclear *NFκB* activity is boosted and the duration of action is significantly shortened, while the influence of changing degradation rates on the duration of action still remains the same. Figure 3b shows the results for low values of the unbinding rate *k*_*soff*_ (0.02 *min*^−1^) : the time courses of both nuclear *NFκB* and synthetic decoy ODNs concentrations are drastically different from those are in Fig. 3a. The duration of action is significantly prolonged in Fig. 3b due to the slow unbinding rate, while the dependence on degradation rates (*λ*_*s*_) of the duration of action remains the same as was found in Fig. 3a.

**Fig. 4:**
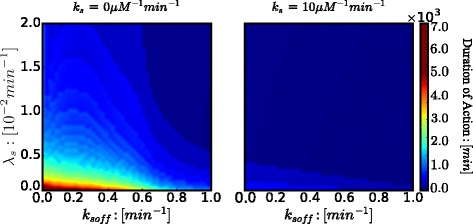
Duration of Action as a function of nuclease-induced degradation (*λ*_*s*_) and unbinding rate (*k*_*soff*_) of *NFκB* synthetic decoy ODNs, under no molecular stripping (*k*_*s*_=0 *μM*^−1^*min*^−1^) and with molecular stripping (*k*_*s*_=10 *μM*^−1^*min*^−1^)

**Fig. 5:**
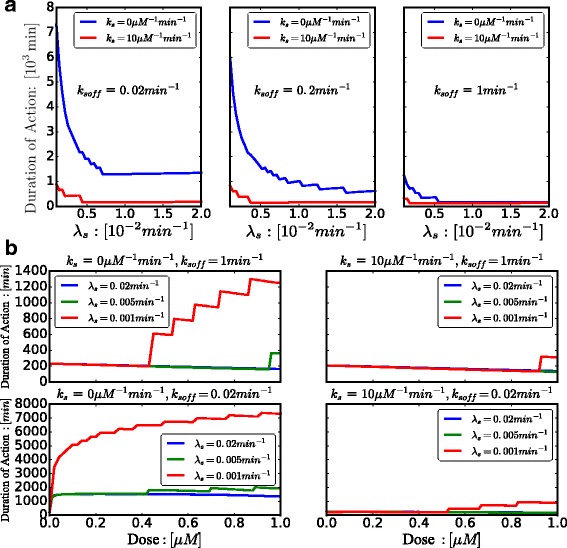
**a** Duration of Action as a function of nuclease-induced degradation rate (*λ*_*s*_) with different unbinding rates (*k*_*soff*_=0.02,0.2,1 *min*^−1^), under no molecular stripping (*k*_*s*_=0 *μM*^−1^*min*^−1^) and with molecular stripping (*k*_*s*_=10 *μM*^−1^*min*^−1^).Dose of *NFκB* synthetic decoy ODNs is set to be 1 *μM*. **b** Duration of Action as a function of dose of *NFκB* synthetic decoy ODNs under different degradation rates (*λ*_*s*_=0.001,0.005,0.02 *min*^−1^), under slow unbinding rate (*k*_*soff*_=0.02 *min*^−1^) and fast unbinding rate (*k*_*soff*_=1 *min*^−1^), under no molecular stripping (*k*_*s*_=0 *μM*^−1^*min*^−1^) and with molecular stripping (*k*_*s*_=10 *μM*^−1^*min*^−1^)

The comparisons of the quantified duration of action between different scenarios are illustrated in Figs. 4 and 5. Figure 4 elaborates on the dependence of the duration of action on the nuclease-induced degradation rate (*λ*_*s*_) and the unbinding rate of *NFκB* synthetic decoy ODNs (*k*_*soff*_). In the absence of molecular stripping, degradation effectively decreases the duration of action. When the unbinding rate is increased, the dependence of duration of action on degradation is attenuated. Also when degradation is slow, a slow unbinding rate leads to longer duration of action, while a fast unbinding rate shortens the duration of action. When degradation is fast, changing unbinding rate *k*_*soff*_ has very little effect on the duration of action. When molecular stripping comes into play, it significantly reduces the duration of action for most of the parameter range and makes the duration of action fairly uniform when varying over a wide range of both the degradation rate (*λ*_*s*_) and the unbinding rate (*k*_*soff*_). These results clearly show that molecular stripping diminishes the therapeutic efficacy of *NFκB* synthetic decoy ODNs for a wide range of parameters. Also, when degradation is very fast, changing the unbinding rate *k*_*soff*_ of synthetic decoy ODNs does not significantly improve therapeutic efficacy.

Figure 5a explicitly illustrates the dependence of the duration of action on the degradation rate (*λ*_*s*_) and on the rate of molecular stripping. When the unbinding rate is very slow (*k*_*soff*_=0.02 *min*^−1^), the duration of action decreases with increasing degradation rate, but further increase of the degradation rate (*λ*_*s*_) above a certain level (∼ 8×10^−3^ *min*^−1^) does not decrease the duration of action. Because of the high binding affinity of *NFκB* synthetic decoy ODNs with slow unbinding rates, there remains a strong inhibitory effect on nuclear *NFκB* activity even when degradation is fast. The situation changes when the unbinding becomes faster (*k*_*soff*_=0.2 *min*^−1^). Degradation monotonically decreases the duration of action due to reduced binding affinity of synthetic decoy ODNs. When unbinding is very fast (*k*_*soff*_=1 *min*^−1^), the duration of action is very short and changing the degradation rate has a very small effect on the duration of action. Overall, our model shows that molecular stripping significantly decreases the duration of action across a wide range of degradation rates.

To analyze the effect of dose on the duration of action, we performed simulations for different doses and degradation rates (*λ*_*s*_) of *NFκB* synthetic decoy ODNs, both when there is molecular stripping(*k*_*s*_=10 *μM*^−1^*min*^−1^) and in the absence of molecular stripping (*k*_*s*_=0 *μM*^−1^*min*^−1^). Nevertheless, the effect of different degradation rates in the duration of action is still noticeable. As illustrated in Fig. 5b, we set the *k*_*soff*_=1 *min*^−1^ to be the fast unbinding rate. It is interesting to see that the duraton of action is more sensitive to dose when degradation is slow (*λ*_*s*_=0.001 *min*^−1^), while when degradation is rapid (*λ*_*s*_=0.02 *min*^−1^) the duration of action becomes relatively insensitive to dose. Figure 5b shows that molecular stripping plays a dominant role in shortening the duration of action, making the dose response curve more insensitive compared with the situation when there is no molecular stripping; nevertheless some influence of changing degradation rates remains. When we set *k*_*soff*_=0.02 *min*^−1^ to be the slow unbinding rate, the dose response curve shows much more sensitive compared with when there is fast unbinding (*k*_*soff*_=1 *min*^−1^) and the duration of action is effectively prolonged, while the effect of degradation rates on dose response remains the same as when unbinding is fast (*k*_*soff*_=1 *min*^−1^). The therapeutic efficacy is a more pronounced function of dose when degradation and unbinding are slow. In this regard, recent studies indicate that the degradation rate can be artificially modulated by attaching protective moieties to the DNA, and the unbinding kinetic rates (*k*_*soff*_) can also be readily tuned in-vitro [38, 39].

## Discussion

The aim of our study is to quantiatively understand the systems pharmacology of *NFκB* synthetic decoy ODNs. We employed sophisticated mathematical models, both deterministic and stochastic, to quantify how synthetic decoy ODNs change the deterministic and stochastic dynamics of relevant species, such as nuclear *NFκB*. We found that molecular stripping blunts the efficacy of synthetic decoy ODNs-mediated inhibition of nuclear *NFκB* activities. More importantly, we also discovered that synthetic decoy ODNs can also change the stochastic dynamics of the regulatory network of *NFκB*.

Beyond the minimal network model analyzed in this paper, we expect that the therapeutic efficacy of *NFκB* synthetic decoy ODNs in vivo will also be modulated by the cellular machinery extrinsic to our network model via coupling to different oscillatory gene circuits. Such coupling would not only change the systems pharmacology of the *NFκB* synthetic decoy ODNs, but also may lead to higher level of temporal organization of the underlying biochemical circuitry. We hope to explore the role of oscillator coupling in the systems pharmacology of synthetic decoy ODNs in a future study. Also, in order to further study their therapeutic efficacy, the mechanisms involved in the optimal delivery of synthetic decoy ODNs into the nucleus need to be elucidated. Nevertheless, quantitative understanding of the dependence of therapeutic efficacy of synthetic decoy ODNs on dose, unbinding kinetic rates and nuclease-induced degradation should help improve therapeutic strategies based on synthetic decoy ODNs.

Another limitation of the current model is the estimation of the kinetic rates of *NFκB* genomic binding sites from Protein Binding Microarray (PBM) data. These data quantify the direct interaction between *NFκB* dimers and short DNA sequences [26]. However, in-vivo transcription factor-DNA interactions are often influenced by chromatin modifications and transcriptional cofactors, which are known to affect the binding/unbinding kinetic rates of genomic sites to *NFκB* [40–43]. For example, the participation of *NFκB* cofactors can turn a “weak” genomic binding site into a “strong” site, simply via the cooperative binding [40, 41]; chromatin modifications change the accessibilty of condensed genomic DNA to transcription factors, which can dramatically change the effective kinetic rates of genomic binding sites to *NFκB* [42, 43]. Incorporating these in-vivo mechanisms into the model might provide a more realistic account on the modulation of the *NFκB* signaling network by synthetic decoy ODNs.

The minimal model developed in this paper generates dynamics similar to the experimental results [20, 23, 44], however, there are several limitations. Firstly, the minimal model is a parsimonious approximation of the full model, the output iof the minimal model might deviate from the that of the full model to a great extent. In order to validate the minimal model, several techniques can be used, such as black-box tests that validate the correctness of the input-output transformations and the sensitivity analysis which validate the model’s behavior under a wide range of parameters and inputs. Secondly, the outputs of the minimal model might contain an estimation bias to the outputs of the full model. There are vast amount of literatures in statistics, operations research and software engineering regarding the validation, verification and uncertainty analysis of the simulation study. Good references include Balci (1994) [45], Wu and Hamada (2009)[46]. In spite of limitations, minimal models can help us identify the key elements governing the entire process. More importantly, compared with the full model, minimal models usually contain far less components and parameters, so they can be used to approximate results from the full model with much less computational cost.

One of the significant findings that stems from our study is that the stochastic dynamics of the system is relatively sensitive to the synthetic decoy ODNs having comparable unbinding rates with that of the *IκB* promoter site (in-resonance synthetic decoy ODNs). This finding might shed light on future studies on single cell gene expression and precision medicine [47–49]. For instance, in-resonance synthetic decoy ODNs could be employed to boost the noise on gene expression levels in cancer stem cells, thus diversifying differentiation pathways. This strategy might be able to facilitate therapies that target cancer stem cell [50].

## Conclusion

In the present work, we report the results from a quantitative model of the systems pharmacology of *NFκB* synthetic decoy ODNs. Our model is based on recently developed systems biology models of *NFκB* signaling networks. Stochastic simulations and deterministic mass action simulations of the quantitative model are able to uncover the dependency of the therapeutic efficacy of *NFκB* synthetic decoy ODNs on dose level, unbinding kinetic rates and their nuclease-induced degradation rate, as well as to predict their influence on the stochastic dynamics of the regulatory network. Our Results show that therapeutic efficacy is inversely correlated to the degradation and unbinding rates of the *NFκB* synthetic decoy ODNs, while being positively correlated with the dose level. More importantly, the stochastic dynamics of the network is heavily influenced by the decoy ODNs having unbinding rates in-resonance with the *IκBα* promoter unbinding kinetic rate. Although it is beyond the scope of this work to elaborate on the toxicology and relevant side effects of *NFκB* synthetic decoy ODNs, our model should provide quantitative guidance for translational researchers to find the “therapeutic window” of *NFκB* synthetic decoy ODNs, in order to optimize their overall safety and therapeutic efficacy.

## Additional files

Additional file 1 Supplementary Materials. (PDF 1263 kb)

Additional file 2 DNA sequences used in the Protein-Binding Microarray (PBM) experiments, and PBM-derived z-scores for all described experiments. (XLSX 1509 kb)
