# Supplementary material for: Modeling the therapeutic efficacy of NFκB synthetic decoy oligodeoxynucleotides (ODNs)
Source: BMC Syst Biol. 2018 Jan 30;12:4. doi: 10.1186/s12918-018-0525-6 (PMC5791368; doi:10.1186/s12918-018-0525-6)
Supplement: Supplementary file 1 — Supplementary Materials. (PDF 1263 kb) [file 12918_2018_525_MOESM1_ESM.pdf]

Supplementary Materials

Zhipeng Wang[1], Davit A Potoyan[1], and Peter G Wolynes[1]

[1] *Center for Theoretical Biological Physics, Department of Chemistry and Department of Physics and Astronomy, Rice University, Houston TX 77005*

1 The Histogram Estimator for the Distribution of DNA sites' Unbinding Rates

We assume the decoy sites' binding free energies (ΔG_b) take a normal distribution, which indicates that $\ln k_{doff} \sim \mathcal{N}(\Delta\hat{G}, \sigma^2)$. We adopt a 15-bin equally-spaced histogram to approximate the distribution of $\ln k_{doff}$ by dividing the distribution into 15 non-overlapping intervals, where each bin represents a decoy site with the corresponding unbinding rate. The historgram estimator enables us to use 15 different decoy species with different unbinding rates to approximate the distribution of unbinding rates of the whole population of decoy sites. Monte Carlo Simulations can be readily performed by treating the whole population of decoy sites as 15 different reacting chemical species, each with the different unbinding kinetic rate determined by the histogram estimator.

The histogram estimator developed by the statistics community[1] also considers further about determining the optimal band width of histogram bins to acheive better goodness-of-fit, as well as the bias-variance tradeoff of statistical estimations. Those are beyond our current focus and need. In this paper we adopted the simple division of the distribution of unbinding rates into 15 bins with the equal band width. We choose 15 bins as it is accurate enough to approximate the distribution while keeping the total number of chemical species in the reacting system relatively small to facilitate reasonably fast Monte Carlo Simulations. If the number of bins increases, the approximation will be more unbiased, but the total number of chemical species in Monte Carlo Simulations will grow thus it will slow down the simulation. Here we choose the appropriate bin numbers to balance out the problem of "curse of dimensionality" and the accuracy of approximations.

2 Comparison of SPR-determined unbinding rate k_{off} and and PBM-determined z scores for p65-p50 heterodimers

In order to convert the z-scores determined by Protein Binding Microarray (PBM) [2] to unbinding rates of DNA binding sequences for p65-p50 heterodimers, we use the data in [2] of unbinding rates (k_{off}) for mice p65-p50 heterodimers determined by Surface Plasmon Resonance (SPR) (six independent SPR measurements). We did the linear regression of $\ln(\ln 2/k_{off})$ over the corresponding z-scores, which is the same as the authors did for c-Rel-c-Rel and p50-p50 homodimers. The linear regression curves and the corresponding R squares and linear regression equation are shown in Figure 1:

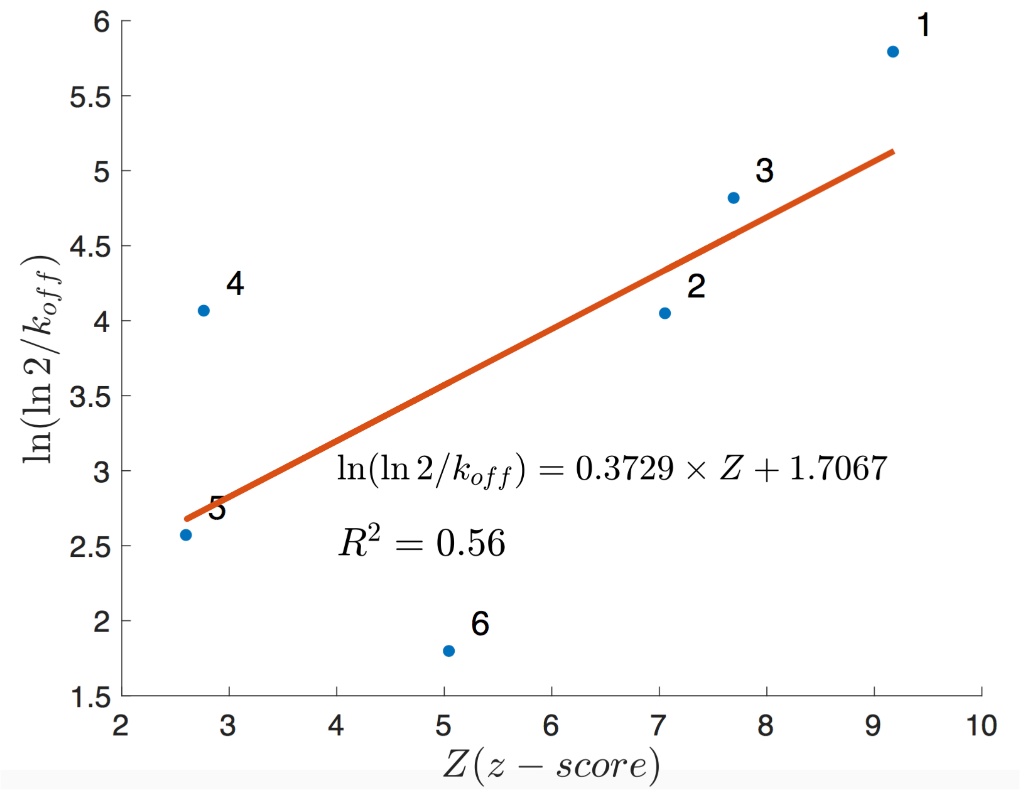

(a)

Figure 1: (a) Linear regression of SPR-determined unbinding rate k_{off} over the PBM-determined z scores for mice p65-p50 heterodimers. Data is determined from six independent measurements (SPR data).

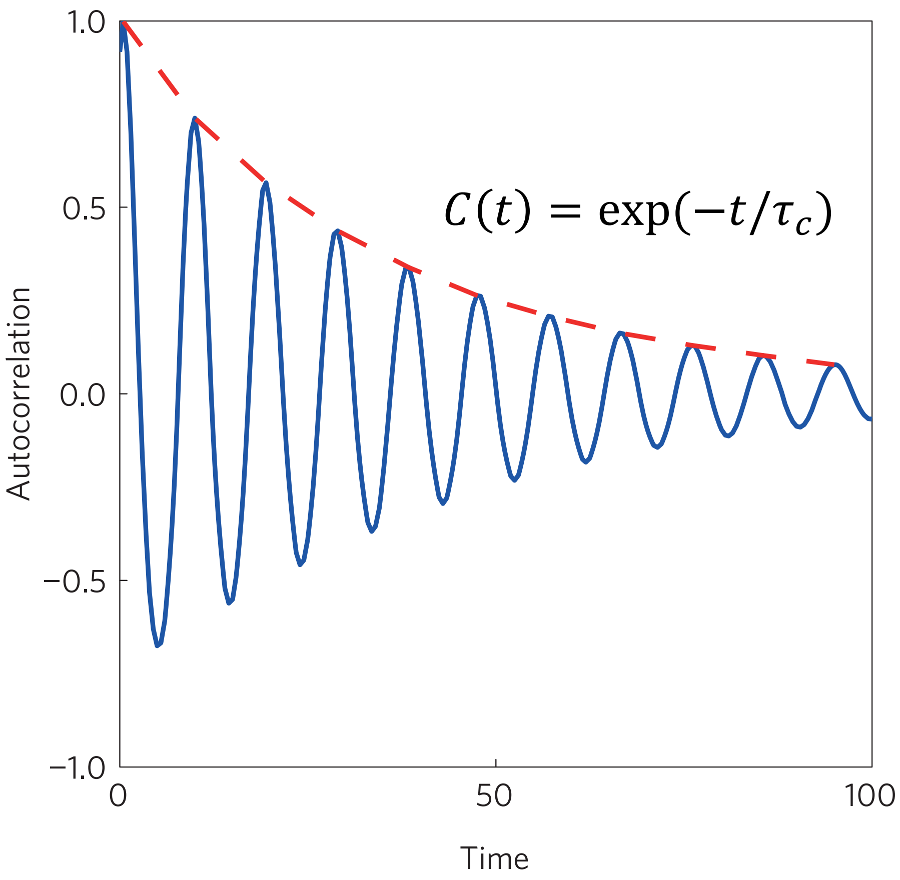

Figure 2: **The instruction of quantifying temporal coherence of stochastic oscillatory dynamics.** In Figure 3, the examplary Periodic Normalized Autocorrelation function is illustrated (plotted in a blue line), and the red dash line represents an exponential decay fitted to the envelope of that autocorrelation function. The oscillation quality thus can be further calculated by τ_c/T in which T is the oscillation period.

References

[1] D.W.Scott. On optimal and data-based histograms. *Biometrika*, 66:605, 1979.

[2] T.Siggers et al. Principles of dimer-specific gene regulation revealed by a comprehensive characterization of NF-κB family DNA binding. *Nature Immunology.*, 107:4016, 2010.
